# Functional capacity and inflammatory biomarkers as predictors for right atrial volume index in COPD patients

**DOI:** 10.1007/s10554-023-02871-5

**Published:** 2023-05-22

**Authors:** Lamiaa Khedr, Naglaa F. Khedr, Rehab H. Werida

**Affiliations:** 1 Department of Cardiology, Faculty of Medicine, Tanta University Hospital, Tanta University, Tanta, Egypt; 2 Biochemistry Department, Faculty of Pharmacy, Tanta University, Tanta, 31527 Egypt; 3 Clinical Pharmacy and Pharmacy Practice Department, Faculty of Pharmacy, Damanhour University, Damanhour, 22514 Egypt

**Keywords:** Adiponectin, COPD, Functional capacity, Hs-CRP, IL-1β, Neopterin, Right atrial volume index

## Abstract

**Objective:**

Chronic obstructive pulmonary disease (COPD) is a leading cause of mortality and right-heart complications. So, this study aimed to evaluate the role of right atrial volume index (RAVI), inflammatory biomarkers and functional capacity in predicting poor outcomes for patients with COPD, classified by COPD assessment test (CAT) questionnaire, as early predictors of right heart diseases.

**Methods:**

151 patients with COPD with ejection fraction (LVEF) > 55% were enrolled and classified according to CAT questionnaire into CAT ≥ 10 (group I) and CAT < 10 (group II). RAVI was calculated using Echocardiography. Assessment of RV systolic function was done by Doppler imaging. Functional capacity parameters were assessed by modified medical research council dyspnea scale (mMRC). IL-1β, adiponectin, hs-CRP and neopterin were evaluated by ELSA kits.

**Results:**

Group I (CAT ≥ 10) had higher RAVI (73.92 ± 21.20 ml/m^2^ vs 22.73 ± 6.24 ml/m^2^, p < 0.001), lower S`tri (0.05 ± 0.01 vs 0.13 ± 0.03 m/s, p < 0.001), lower tricuspid annular plane systolic excursion (TAPSE) (1.20 ± 0.17 cm *vs* 2.17 ± 0.48 cm, p < 0.001), higher RVSP (54.88 ± 7.97 vs 26.79 ± 9.84 mmHg, p < 0.001) compared with group II (CAT < 10). RAVI was good predictor of CAT (r = 0.954, p < 0.001) and strongly correlated with tricuspid S`tri, RVSP, tricuspid E/e′ and Mitral E/e′ (r = −0.737, r = 0.753, r = 0.817 and r = 0.515, respectively, p < 0.001). RAVI was correlated with TAPSE (r = −0.673, p < 0.001) and with tricuspid E/A ratio & LVEF (r = 0.628, r = −0.407, respectively, p < 0.001). Hs-CRP: 2.50 ± 1.43 vs 2.03 ± 1.19, IL-1β: 37.96 ± 14.35 vs 27.57 ± 8.06, neopterin: 91.37 ± 17.30 vs 76.90 ± 16.75, p < 0.05) were significantly higher besides lower adiponectin levels (3.19 ± 1.98 vs 5.32 ± 1.33 p < 0.05) in group I as compared to group II.

**Conclusion:**

Functional capacity might be useful predictor for right heart diseases in COPD patients. Inflammatory biomarkers, low adiponectin and high Hs-CRP, IL-1β and neopterin levels, might not only be useful to monitor treatment response but may also help to discriminate patients with a worsen prognosis.

**Supplementary Information:**

The online version contains supplementary material available at 10.1007/s10554-023-02871-5.

## Introduction

Globally, chronic obstructive pulmonary disease (COPD) is one of the leading cause of illness and mortality. COPD is the world's fourth leading cause of death, with significant rises in frequency and mortality expected in the future [1]. COPD is classified as a systemic condition, which has been recognized in the most recent COPD guidelines. One such systemic aspect is the risk of cardiovascular disease. As a result, establishing a reliable method of identifying patients at increased risk is critical [2].

Systemic effects and extrapulmonary comorbidities are common and significantly impact health outcomes in patients, including mortality, cardiovascular diseases, musculoskeletal disorders, diabetes mellitus type II and metabolic syndrome are among the most prevalent comorbidities; however the underlying molecular basis linking COPD and comorbidities are still not fully understood [3–5].

The functional and anatomical relationship between the lungs and the heart is such that any dysfunction of one of them is likely to have consequences on the other [6]. This interaction is important in patients with COPD and can be summarized in two types of association. First, one that relates pathologies that share similar risks, such as cigarette smoke and coronary artery disease (CAD), or congestive heart failure and COPD; and secondly, those that result in dysfunction of the heart from primary lung disease, such as secondary pulmonary hypertension and ventricular dysfunction due to increased intra-thoracic mechanical loads [6].

COPD can cause right-heart complications, such as concentric right ventricular (RV) hypertrophy and raised RV end-diastolic pressures, which are the initial signs of high pulmonary artery pressure, followed by RV systolic dysfunction. The end-stage COPD patients frequently experience RV dysfunction [7].

The chronicity of the systemic inflammation associated with COPD is maintained by increased production of several pro-inflammatory cytokines at both the serum and airway levels. High sensitivity C-reactive protein (Hs-CRP), fibrinogen, interleukin-1 (IL-1), IL-10, monocyte chemoattractant protein-1 (MCP1), IL-8, and IL-6 have all been linked to the disease development and exacerbation [8–10].

Hs-CRP levels may be useful biomarkers in monitoring COPD and asthma response to treatment during an exacerbation episode. However, the anti-inflammatory cytokine IL-10 has been shown to have an inverse relationship with COPD. COPD patients’ metabolic abnormalities have also been linked to a systemic inflammatory state [3, 11, 12]. Recent clinical investigations have highlighted the role of metabolic syndrome biomarkers to predict lung function impairment [13].

Recent studies have emphasized the importance of adiponectin and its receptors in obesity, metabolic syndrome, insulin resistance, hyperinsulinemia and type 2 diabetes, as well as with cardiovascular diseases [14, 15]. Adiponectin appears involved in the development and progression of several local and systemic inflammatory processes [16]. Moreover, adiponectin appears to be an appealing biomarker in COPD, and it represents a promising disease indicator with possible treatment implications [17]. Adiponectin appears to have a direct protective effect on lung epithelial A549 cells, including anti-proliferative and anti-inflammatory properties. Adiponectin also protects A549 lung cells from cytotoxicity caused by TNF-α or IL-1. Elevated serum IL-1β and IL-17 levels may be used as a biomarker for indicating persistent neutrophilic airway inflammation and potential ongoing exacerbation of COPD [18]. Hypoadiponectinemia was detected in asthma and COPD during all stages of the diseases [19, 20].

Neopterin is strongly predictive of significant adverse cardiac events in angina [21] and is a recognized risk factor for acute respiratory tract infections in COPD patients [22]. Recent studies have looked at how chronic low-grade inflammation affects health status, disability, and activity tolerance; they found that COPD patients with increased CRP had worse quality of life, activity tolerance, and lung function [23, 24]. Other research shows that grip strength predicts mortality after pneumonia as well as neopterin as a possible contributor to respiratory infections [25].

Whether or not systemic inflammation in COPD is fully known, more research needs to be done on the causes of systemic involvement. So, this study was set out to find whether the right atrial volume index (RAVI), inflammatory biomarkers, and functional capacity in COPD patients who categorized by the COPD assessment test (CAT) questionnaire could be used as an early predictor of right heart diseases.

## Patients and methods

This observational study included one-hundred and fifty-one COPD patients, who were selected from patients visited Chest Department and Cardiology Department or attending Chest outpatient clinic of Tanta University Hospitals, Tanta, Egypt. The study was done in the period between January 2022 to August 2022. The study was approved (No. 422PP49) by the Research Ethics Committee, Faculty of pharmacy, Damanhour University, Damanhour, Egypt, and Tanta University Hospital, Egypt; according to the Helsinki Declaration of 1964, as revised in 2013. The study protocol was registered at clinicaltrials.gov (NCT05366400). All participants signed an informed consent to participate in the study.

### Inclusion criteria

The COPD patients were diagnosed based on clinical data and validated by spirometry findings, according to the diagnostic criteria of the GOLD guidelines for chronic obstructive pulmonary disease [26]. Patients were included if they were over the age of 18y and had a left ventricular ejection fraction (LVEF) > 55%. Their demographic characteristics (age and gender) were documented. Active smoking within the previous 12 months was considered as current cigarette smoking. History or previous medical data were used to record coronary artery disease (Table 1).

**Table 1:** Demographic and clinical data of the studied groups

	Group I (CAT) ≥ 10 (n = 60)	Group II (CAT) < 10 (n = 91)	P value
Age (years)	59.93 ± 10.62	59.79 ± 10.79	0.936
Gender (F /M) (%)	14/46 (23.3/76.7%)	15/76 (16.5/83.5%)	0.296
BMI (kg/m^2^)	28.80 ± 4.71	28.46 ± 4.84	0.666
Hemoglobin (mg/dL)	12.33 ± 0.91	13.17 ± 1.22	0.000
HbA1C %	6.35 ± 0.74	6.68 ± 0.80	0.016
Total cholesterol (TC, mg/dL)	192.07 ± 19.92	198.36 ± 25.96	0.095
Serum creatinine (mg/dL)	0.99 ± 0.14	1.08 ± 0.25	0.003
Systolic BP (mmHg)	134.13 ± 7.24	134.51 ± 7.43	0.760
Diastolic BP (mmHg)	81.77 ± 3.66	81.32 ± 3.29	0.445
**Respiratory function**			
FVC (L)	2.90 ± 0.33	3.27 ± 0.48	0.000
FEV1 (L)	1.47 ± 0.12	1.78 ± 0.20	0.000
FEV1/FVC ratio	0.51 ± 0.07	0.55 ± 0.08	0.001
PaO_2_, mmHg	70.48 ± 3.89	79.9 ± 5.60	0.000
**QRS**			
Normal	22(36.7%)	41 (45.1%)	0.350
LBBB	26 (43.3%)	39 (42.9%)	
RBBB	12 (20%)	11 (12.1%)	
**Rhythm**			
Sinus	47 (78.3%)	78 (85.7%)	0.240
AF	13 (21.7%)	13(14.3%)	
**mMRC**			
Class I	21 (35.0%)	37(40.7%)	0.572
Class II	17 (28.3%)	28 (30.8%)	
Class III	22 (36.7%)	26 (28.6%)	
DM	35 (58.3%)	39 (42.9%)	0.063
HTN	28 (46.7%)	45 (49.5%)	0.738
CAD	18 (30%)	23 (25.3%)	0.523
Smoker	35 (58.3%)	59 (64.8%)	0.420
FH	27 (45%)	44 (48.4%)	0.686
ACEIs	24 (40%)	27 (29.7%)	0.189
ARBs	22 (36.7%)	40 (44%)	0.373
CCB	8 (13.3%)	6 (6.6%)	0.162
Statin	18 (30%)	23 (25.3%)	0.523
BB	7 (11.7%)	10 (11%)	0.897

Data presented as mean ± SD or frequency (percentage) as appropriate

*CAT* COPD assessment test, *F* Female, *M* Male, *BMI* body mass index, *HbA1C %* glycated hemoglobin percent, *BP* blood pressure, *FV*C forced vital capacity, *FEV*_*1*_ forced expiratory volume in 1 s, *PaO*_*2*_ partial pressure of oxygen in arterial blood, *LBBB* left bundle branch block, *RBBB* right bundle branch block, *AF* atrial fibrillation, *mMRC* modified medical research council, *DM* diabetes mellitus, *HTN* hypertension, *CAD* coronary artery disease, *FH* familial hypercholesterolemia, *ACEI* angiotensin converting enzyme inhibitor, *ARB* angiotensin receptor blocker, *CCB* calcium channel blocker, *BB* beta-blocker

*Data analyzed using independent T-test or chi-square as appropriate, statistically significance at p < 0.05

### Exclusion criteria

Patients diagnosed with other respiratory diseases (e.g., asthma, interstitial pulmonary fibrosis, tuberculosis or lung cancer), ischemic heart disease, congestive heart failure, valvular heart disease and congenital heart diseases were excluded. Other chronic diseases, such as kidney or liver failure and cancer were also excluded.

### Data collection

Transthoracic echocardiography (TTE) and functional capacity assessment were performed in all patients. Detailed medical history and clinical assessment were obtained from all patients, whom routine biochemical tests were also performed, including hemoglobin, total cholesterol, serum creatinine, blood pressure, and spirometry data. Body mass index (BMI) was calculated from weight and height measurements (BMI = weight (kg)/height (m^2^).

### Echocardiographic examination

All patients underwent a complete echo Doppler study, including two-dimensional (2D), color- flow Doppler and tissue Doppler imaging (TDI) using vivid 9–dimension machine (GE Vingmed, Horten, Norway) at the Cardiology Department (Tanta University Hospitals, Tanta, Egypt). All acquisitions were performed using a broad band M4s transducer according to the recommendations of the American Society of Echocardiography. Imaging was performed while the patient in the left lateral position using standard para-sternal and apical views and two investigators performed the echocardiogram.

Right atrial volume was calculated using biplane area length method using four chamber- views twice. Planimetry of the maximal right atrium area at end-ventricular systole just before opening of the tricuspid valve (at the end of T-wave) from the lateral aspect of the tricuspid annulus, following the RA endocardium, to the septal aspect, excluding the area between the leaflets and annulus, superior and inferior vena cave and RA appendage. The length of the RA was taken as a perpendicular line measured from the middle of the plane of tricuspid annulus to the superior wall of the RA [27]. RA volume was calculated based on the formula: right atrial volume (RAV) (mL) = 0.85 (A^2^)/ L; where *A* is the area in the four-chamber view and *L* is the length of vertical long-axis, as described by Ebtia et al. [27].

The right atrial volume index (RAVI) was derived by dividing the RAV by body surface area, which was calculated using the Du Bois formula [28].

Assessment of the right ventricular systolic function using Tissue Doppler Imaging (TDI)- derived tricuspid lateral annular systolic velocity (S`_tri_), by pulsed wave TDI (m/sec) obtained from the apical 4 chamber view and by tricuspid annular plane systolic excursion (TAPSE), by two-dimensional difference of end-diastolic and end-systolic lines (in cm).

Right ventricular systolic pressure was estimated by calculating the maximal velocity of the tricuspid regurgitant jet and then, using the Bernoulli equation, adding to this value an estimated right atrial pressure based on both the size of the inferior vena cava and the change in diameter of this vessel during respiration. Pulsed Doppler echocardiography for the assessment of the standard diastolic filling velocities of both ventricles was performed using the apical four-chamber view. Thus, the peak early diastolic filling velocity (E-wave) and peak late diastolic filling velocity (A-wave) were recorded, also annular velocities examination used for assessment diastolic function of both ventricles by placing pulsed wave -TDI sample volume at the level of the lateral annulus. Three tissue velocities were recorded, S`- systolic velocity, E`- Early diastolic velocity and A`- Late diastolic velocity.

### Assessment of functional capacity in studied patients

For the assessment of the patients’ functional capacity, who classified by COPD assessment test (CAT) [29], modified medical research council dyspnea scale (mMRC) [30] was used as the following: Grade 0: The patient only gets breathless with strenuous exercise. Grade 1: The patient gets short of breath when hurrying on level ground or walking up a slight hill. Grade 2: The patient walks slower than people of the same age on level ground because of breathlessness or must stop for breath when walking at his own pace on the level. Grade 3: The patient stops for breath after walking about 100 yards or after a few minutes on the level. Grade 4: The patient is too breathless to leave the house, or breathless when dressing.

### COPD assessment test (CAT)

CAT is an eight-item questionnaire with a six-item Likert scale ranging from 0 to 5. The score ranges from zero (completely asymptomatic) to 40 (extremely symptomatic). A CAT score ≥ 10 is associated with a significantly impaired health status [29] (Supplementary Figure).

### Specimen collection and measurements of serum biomarkers

Venous blood samples were withdrawn from each patient between 8 and 9 am after a 30 min rest in the supine position, after 10-12 hour overnight fasting, into serum vacutainer test tubes, at the beginning of the study and after 3 months of intervention and follow up. Blood samples were allowed to clot for 15–30 min then centrifuged at 3000 rpm for 15 min using Hettich Zentrifugen EBA 20 (Merck, Germany). Serum was frozen at −80 °C till biochemical assay of IL-1β, adiponectin, hs-CRP and neopterin using commercially available ELISA kits (Sunred biological technology, Shanghai) according to manufacturer`s instructions. Serum glycated hemoglobin (HbA1c) was determined by ion exchange method using kits obtained from Stanbio Laboratory Company (USA). Total cholesterol and serum creatinine were determined calorimetrically using kits obtained from Biodiagnostic, Giza, Egypt. All samples were measured in duplicate.

### Statistical analysis

The collected data were analyzed using software statistical computer package SPSS version 24.0 (SPSS Inc, Chicago, IL, USA). The normality of each variable’s data was checked using Kolmogorov–Smirnov and Shapiro–Wilk tests. Continuous variables were expressed as mean ± SD. Independent t-test was used to determine the difference between the two groups. Categorical variables are presented as number or percentage and were analyzed using the Chi-Square test to compare the two cohorts. Correlation between variables was evaluated using Pearson’s correlation coefficient. The significance level was set at P < 0.05. Predictors were identified with binary logistic regression using the stepwise method. A significant difference was defined as P value < 0.05.

## Results

**Fig. 1:**
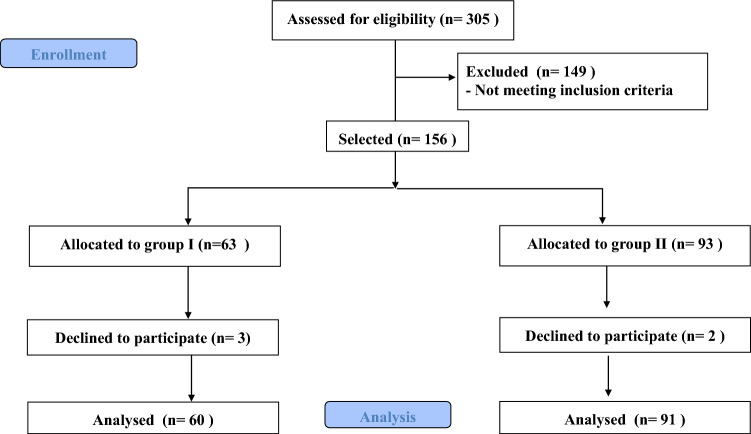
Participants enrollment flow chart

The study included 151 patients with COPD in the final analysis. Patients were divided into two groups according to CAT score, 60 patients (39.74 %) had CAT score ≥ 10 and 91 patients (60.26 %) had CAT score < 10 (Fig. 1).

Table 1 shows that patients with CAT ≥ 10 had a higher prevalence of male gender (76.7%) vs (23.3%) female gender, higher prevalence of atrial fibrillation (21.7% versus 14.3%), higher incidence of advanced mMRC class III (36.7% versus 28.6%) compared to patients with CAT < 10. Other demographic and clinical data of the studied patients presented in (Table 1).

**Table 2:** Measured echocardiographic parameters of the studied groups

	Group I (CAT) ≥ 10, n = 60	Group II (CAT) < 10, n = 91	*P value
RAVI (ml/m^2^)	73.92 ± 21.20	22.73 ± 6.24	< 0.001*
S`tri (m/s)	0.05 ± 0.01	0.13 ± 0.03	< 0.001*
TAPSE (cm)	1.20 ± 0.17	2.17 ± 0.48	< 0.001*
RVSP (mmHg)	54.88 ± 7.97	26.79 ± 9.84	< 0.001*
LVEF (%)	63. 62 ± 4.52	64.63 ± 5.08	< 0.001*
Mitral E/e`ratio	14.01 ± 4.89	8.38 ± 4.67	< 0.001*
Tricuspid E/A ratio	1.90 ± 0.77	0.93 ± 0.32	< 0.001*
Tricuspid E/e` ratio	8.06 ± 0.81	3.82 ± 0.99	< 0.001*

Data presented as mean ± SD

*CAT* COPD assessment test, *RAVI* right atrial volume index, *S`tri* systolic velocity of lateral tricuspid annulus, *TAPSE* tricuspid annular plane systolic excursion, *RVSP* right ventricular systolic pressure, *LVEF* left ventricular ejection fraction, *Mitral E/e`* ratio between early mitral inflow wave/mitral annular early diastolic velocities, *Tricuspid E/A* ratio between tricuspid early/late diastolic velocities, *Tricuspid E/e`* ratio of tricuspid early diastolic wave /tricuspid annular early velocities

*Independent T-test statistically significant at p < 0.05

**Fig. 2:**
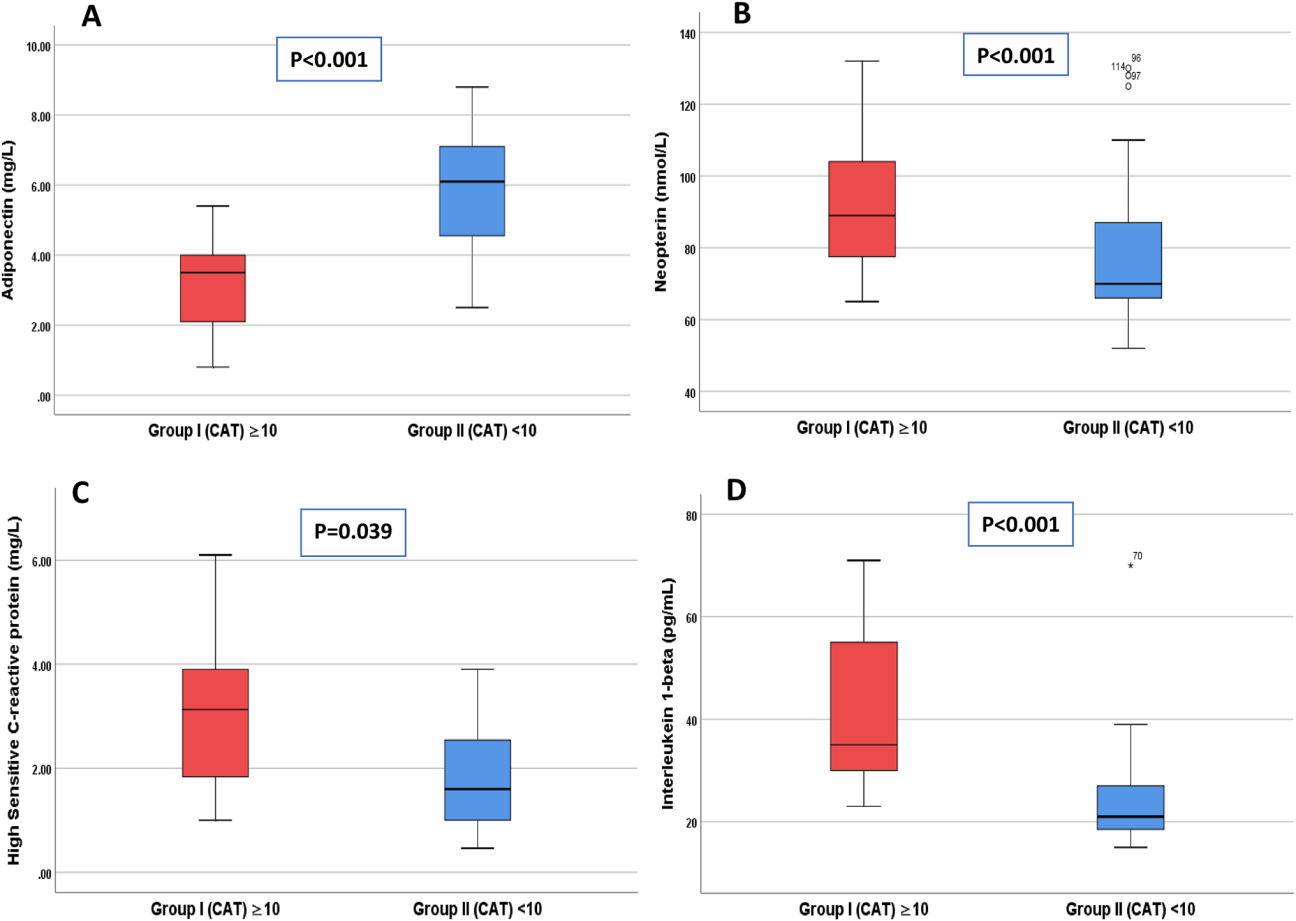
Serum level of adiponectin (A), neopterin (B), high-sensitive C-reactive protein (C), Interleukin-1β (D) in studied groups, CAT=COPD assessment test. Box-and-Whisker plots show the upper and lower quartiles and range (box), median value (horizontal line inside the box), and full range distribution (Whisker line). Independent T-test was used to evaluate the statistical significance.

Patients with CAT ≥ 10 also showed higher RAVI (73.92 ± 21.20 vs 22.73 ± 6.24, P value < 0.001), higher RVSP (54.88 ± 7.97 *vs* 26.79 ± 9.84, P value < 0.001), higher mitral E/e` ratio (14.01 ± 4.89 *vs* 8.38 ± 4.67, P value < 0.001), higher tricuspid E/A ratio (1.90 ± 0.77 *vs* 0.93 ± 0.32, P value < 0.001), higher tricuspid E/e` ratio (8.06 ± 0.81 *vs* 3.82 ± 0.99, P value < 0.001), and lower TAPSE (1.20 ± 0.17 *vs* 2.17 ± 0.48, P value < 0.001), lower S`tri (0.05 ± 0.01 *vs* 0. 0.13 ± 0.03, P value < 0.001), and lower LVEF % (63.62 ± 4.52 *versus* 64.63 ± 5.08, P < 0.001) when compared to patients with CAT < 10 (Table 2). Inflammatory biomarkers (Table 3), Hs-CRP, IL-1β and neopterin were significantly higher (Hs-CRP; 2.50 ± 1.43 vs 2.03 ± 1.19, IL-1β; 37.96 ± 14.35 vs 27.57 ± 8.06, neopterin; 91.37 ± 17.30 vs 76.90 ± 16.75) with lower adiponectin level (3.19 ± 1.98 vs 5.32 ± 1.33 p < 0.05) in group I as compared to group II (Fig. 2).

**Table 3:** Measured inflammatory biomarkers of the studied groups

	Group I (CAT) ≥ 10, n = 60	Group II (CAT) < 10, n = 91	*P value
Hs-CRP (mg/L)	2.50 ± 1.43	2.03 ± 1.19	0.039*
IL-1β (pg/mL)	37.96 ± 14.35	27.57 ± 8.06	< 0.001*
Adiponectin (mg/L)	3.19 ± 1.98	5.32 ± 1.33	< 0.001*
Neopterin (nmol/L)	91.37 ± 17.30	76.90 ± 16.75	< 0.001*

Data presented as mean ± SD

*hsCRP* highly sensitive C-reactive protein, *IL-1β* interleukin 1 beta

*Independent T-test statistically significant at p < 0.05

### Correlation study

Table 4 showed that CAT had a strong positive correlation with RAVI (r = 0.954, p < 0.001), RVSP (r = 0.733, p < 0.001), and tricuspid E/e` (r = 0.685, p < 0.001), tricuspid E/A ratio (r = 0.451, p < 0.001), mitral E/e` (r = 0.553, p < 0.001), hs-CRP (r = 0.345), IL-1- β (r = 0.607), and neopterin (r = 0.312) and a negative correlation with tricuspid S`tri (r = -0.701, p < 0.001), TAPSE (r = −0.693, p < 0.001), and adiponectin (r =−0.854).

**Table 4:** Correlation between CAT score with the echocardiographic variables and measured biomarkers

Variables	CAT Score	
	r	p
RAVI	0.954	< 0.001*
Tricuspid S`tri	−0.701	< 0.001*
RVSP	0.733	< 0.001*
Tricuspid E/e`	0.685	< 0.001*
Mitral E/e`	0.553	< 0.001*
TAPSE	−0.693	< 0.001*
Tricuspid E/A ratio	0.451	< 0.001*
LVEF %	−0.426	< 0.001*
Hs-CRP (mg/L)	0.345	< 0.001*
IL-1β (pg/mL)	0.607	< 0.001*
Adiponectin (mg/L)	−0.854	< 0.001*
Neopterin (nmol/L)	0.312	< 0.001*

*CAT* COPD assessment, *RAVI* right atrial volume index, *S`tri* systolic velocity of tricuspid valve annulus, *RVSP* right ventricular systolic pressure, *Mitral E/e`* mitral early/mitral annular early velocities ratio, *TAPSE* tricuspid annular plane systolic excursion, *LVEF* left ventricular ejection fraction, *Tricuspid E/A* tricuspid early/late velocities ratio, *Tricuspid E/e`* tricuspid early/tricuspid annular early velocities ratio, *hsCRP* highly sensitive C-reactive protein, *IL-1β* interleukin 1 beta

*R* Person correlation

Table 5 showed that RAVI had a strong positive correlation with RVSP (r = 0.753, p < 0.001), tricuspid E/e`(r = 0.817, p < 0.001), mitral E/e` (r = 0.515, p < 0.001), tricuspid E/A ratio (r = 0.628, p < 0.001), Hs-CRP (r = 0.485), IL-1-β (r = 0.691), and neopterin (r = 0.299) and a negative correlation with tricuspid S`tri (r = −0.737, p < 0.001), TAPSE (r = −0.673, P < 0.001), LVEF (r = −0.407, p < 0.001) and adiponectin (r =−0.833, p < 0.001). Table 6 shows the results of binary logistic regression analysis of the measured inflammatory biomarkers in the studied groups. The analysis's findings demonstrated that high levels of IL-β and neopterin and low level of adiponectin were associated with high CAT score and so may predict poor outcomes in COPD patients.

**Table 5:** Correlation between RAVI with the echocardiographic variables and measured biomarkers

Variables	RAVI	
	r	p
Tricuspid S`tri	−0.737	< 0.001*
RVSP	0.753	< 0.001*
Tricuspid E/e`	0.817	< 0.001*
Mitral E/e`	0.515	< 0.001*
TAPSE	−0.673	< 0.001*
Tricuspid E/A ratio	0.628	< 0.001*
LVEF %	−0.407	< 0.001*
Hs-CRP (mg/L)	0.485	< 0.001*
IL-1β (pg/mL)	0.691	< 0.001*
Adiponectin (mg/L)	−0.833	< 0.001*
Neopterin (nmol/L)	0.299	< 0.001*

*RAVI* right atrial volume index, *S`tri* systolic velocity of tricuspid valve annulus, *RVSP* right ventricular systolic pressure, *Mitral E/e`* mitral early/mitral annular early velocities ratio, *TAPSE* tricuspid annular plane systolic excursion, *LVEF* left ventricular ejection fraction, *Tricuspid E/A* tricuspid early/late velocities ratio, *Tricuspid E/e`* tricuspid early/tricuspid annular early velocities ratio, *hsCRP* highly sensitive C-reactive protein, *IL-1β* interleukin 1 beta

*R* Person correlation

**Fig. 3:**
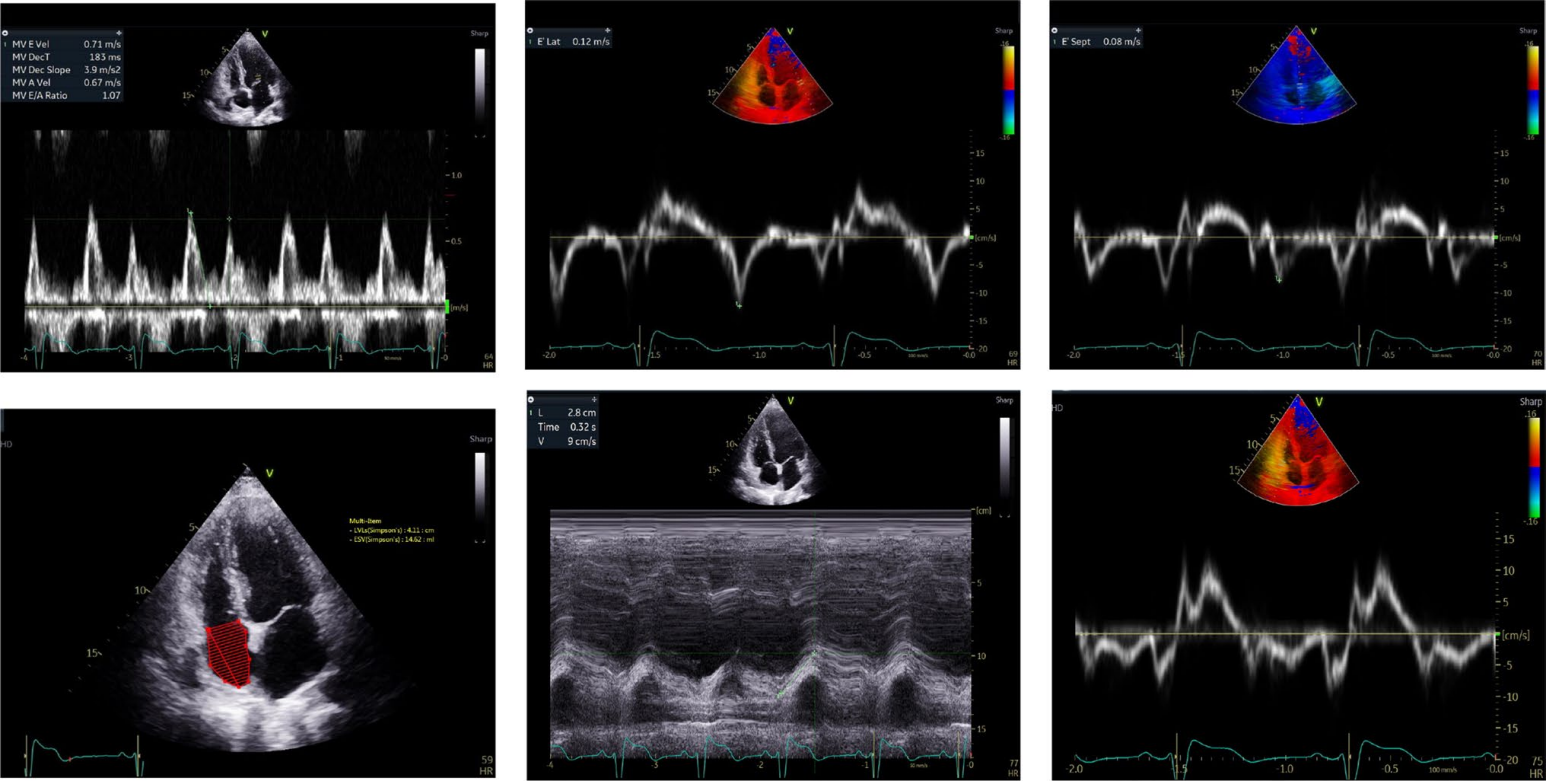
Echocardiographic Assessment of studied patients with CAT score <10

Echocardiographic assessment of studied patients with CAT score < 10 showed normal diastolic function and left ventricular filling pressure (average 7.2), normal RV systolic function (TAPSE = 2.8 cm), tissue doppler S wave velocity 12 (cm/s) and normal RA volume index Fig. 3.

**Fig. 4:**
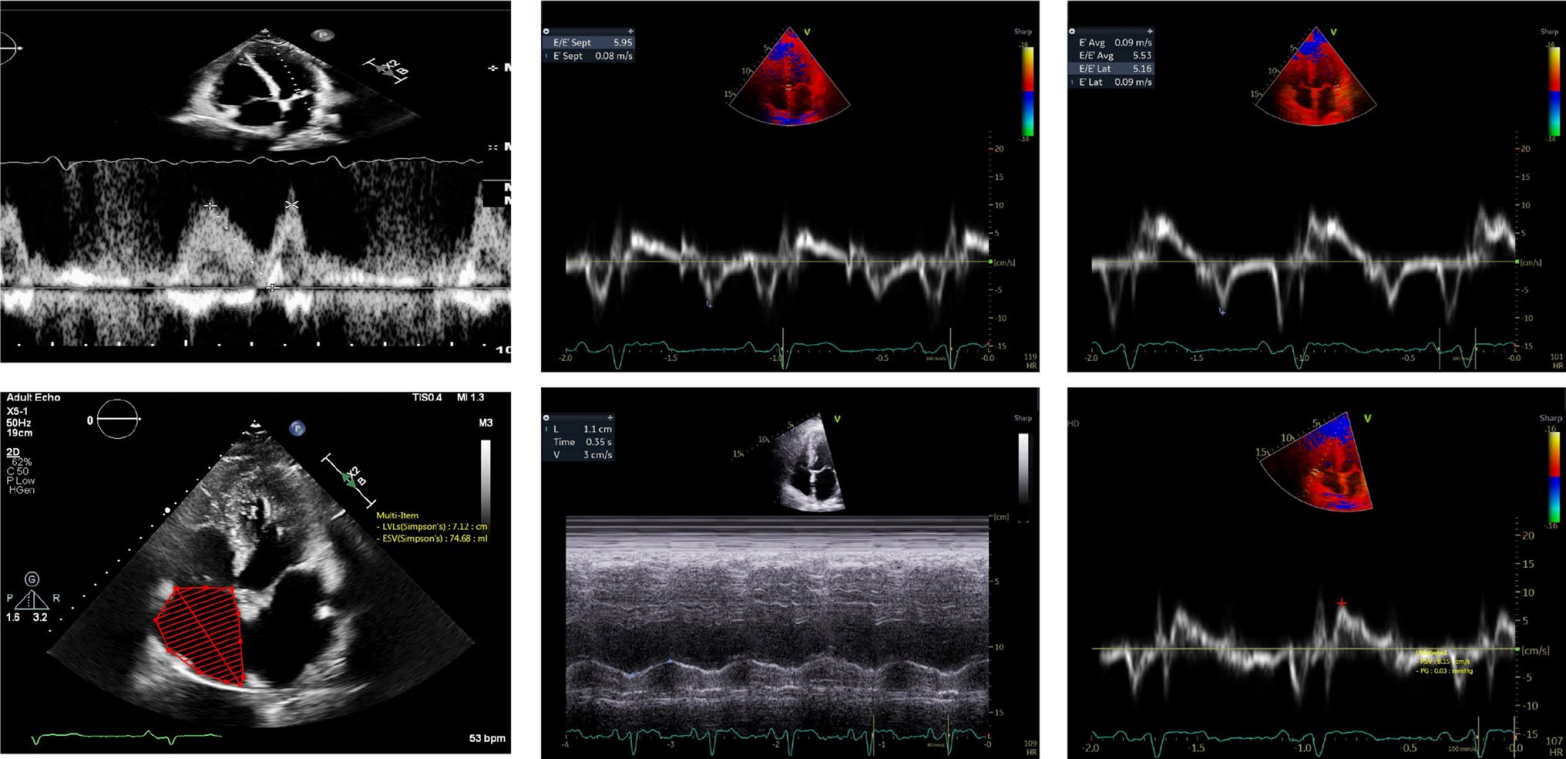
Echocardiographic Assessment of studied patients with CAT score ≥10

Echocardiographic assessment of studied patients with CAT score ≥ 10 shows Grade I diastolic disfunction, impaired RV systolic function (TAPSE = 1.1 cm) tissue doppler S wave velocity 8.15 (cm/s) and increased RA volume index Fig. 4.

## Discussion

The present study aims to evaluate the relationships between right atrial volume index, functional capacity and inflammatory biomarkers in COPD patients.

Up till recently, the routine transthoracic echocardiogram of evaluation of RV function has been neglected. This neglect has been caused in part by challenges with visualizing and evaluating the RV quantitatively. Furthermore, the accepted standards for quantifying both systolic and diastolic RV dysfunction are still not well established. In patients with congestive HF, RV systolic function is now becoming a significant predictor of morbidity and mortality [31–33].

However, precise echocardiographic assessment of RV systolic function is challenging, primarily because the morphology of RV is complicated. The ability to visualize the RA allows a quantitative, highly reproducible assessment of the right atrial volume that can be indexed to body surface area (RAVI). RA volume can serve as a quantitative marker of RV dysfunction severity [34, 35].

In the present study, COPD patients presented with worse CAT were assessed by echocardiography. The results of this study confirmed the initial hypothesis that RAVI is associated with RV systolic and diastolic dysfunction in patients with COPD. Elevated RAVI showed a strong correlations with RV systolic function parameters, such as systolic velocity of the tricuspid annulus and TAPSE, as well as systolic pulmonary artery pressure (sPAP).

In the present study, RAVI was higher in patients with a low functional capacity (CAT score ≥ 10) than those with CAT score < 10. Although the current study showed that CAT score had a strong correlation with systolic velocity of tricuspid valve annulus (S`_tri_) and estimated RVSP, also correlation with LV ejection fraction, TAPSE and mitral E/e` but in a weaker manner. However, RAVI was the best predictor of CAT. Unfortunately, research involving this parameter is scarce at this point, leaving serious gaps in information.

Previous study by Mantziari et al. [36] reported that RAVI, TAPSE and reduced LVEF are independent predictors of functional capacity in patients with chronic HF but RAVI was a stronger predictor of functional capacity assessed by Duke Activity Status Index (DASI) in patients with stable chronic heart failure with high sensitivity and specificity in the subgroup of patients had impaired RVSF (79% and 90% respectively) in predicting DASI < 10. They found also RAVI was the single independent predictor of low DASI (beta =−0.501, p < 0.001).

Sallach et al. [34] who studied the relationship between RAVI and right ventricular (RV) systolic and diastolic function, as well as long-term prognosis in patients with chronic systolic heart failure (HF), showed that the increasing tertiles of RAVI was predictive of HF hospitalization and remained an independent predictor of adverse clinical events in patients with chronic stable heart failure after adjusting for age, brain naturetic peptide, LVEF, RV systolic dysfunction stage, and tricuspid E/e` ratio. Also, they showed that RAVI ≥ 41.6 ml/m^2^ (optimal ROC cutoff) had 68% sensitivity and 92% specificity for predicting New York Heart Association (NYHA) functional class ≥ III.

Darahim et al. [37], who investigated the relation between RAVI and the prognosis of patients with chronic systolic HF. They demonstrated that, RAVI is an independent predictor of adverse outcomes with a threshold value of 29 mL/m^2^. Patients who experienced adverse events had a greater mean RAVI (45.5 ± 15 mL/m^2^ vs. 25.2 ± 11 mL/m^2^, P < 0.001) than healthy subjects.

According to earlier study by Hamdan et al. [38]**, **the mitral E/e' ratio was associated with hospitalization for worsening heart failure in individuals with substantial LV dysfunction. These studies looked at the association between mitral annular velocities and clinical symptoms.

**Table 6:** Binary logistic regression analysis of measured inflammatory biomarkers of studied groups

Variables in the equation								95% CI for Exp(B)	
		B	S.E	Wald	df	Sig.	Exp(B)	Lower	Upper
Step 1^a^	Hs-CRP (mg/L)	0.729	0.434	2.814	1	0.093	2.072	0.885	4.854
	IL-1β (pg/mL)	−0.236	0.069	11.826	1	0.001	0.790	0.691	0.904
	Neopterin (nmol/L)	−0.079	0.025	9.682	1	0.002	0.924	0.879	0.971
	Adiponectin (mg/L)	1.486	0.349	18.088	1	0.000	4.420	2.228	8.768
	Constant	5.791	2.882	4.038	1	0.044	327.224		

*CI* confident interval

^a^Variable(s) entered on step 1: hs-CRP: high sensitive C-reactive protein (mg/L), interleukin 1-beta (pg/mL), neopterin (nmol/L), adiponectin (mg/L).

In the present study, RV systolic function was evaluated using the tricuspid annular plane systolic excursion (TAPSE) and the peak systolic velocity of the tricuspid valve annulus (S'tri). Between RAVI, S'tri, and TAPSE, a negative association was found, in this instance these findings are similar to those of Sallach et al. [34]**,** who classified RV systolic function in patients as normal, mild, moderate, moderate/severe, and severe using TAPSE and S'tri to assess RV systolic dysfunction. RAVI markedly increased with worsening RV systolic dysfunction. Additionally, both in the univariable and multivariable analyses, a significant correlation between RAVI and RV systolic dysfunction was discovered (r = 0.61 and 0.38, respectively; p = 0.0001 for both) [34]. Moreover, Mantziari et al. [36] reported that RAVI had a strong correlation with right ventricular systolic function (RVSF) parameter (TAPSE) (r =−0.47, p = 0.001) and RV end-systolic diameter (r =−0.48, p = 0.002).

In addition, RV fractional area change (RVFAC) and peak systolic velocity were assessed using tissue Doppler imaging at the tricuspid annulus (S`_tri_) that showed a good correlation between RAVI and worsening S`tri and RVFAC (r =−0.59, r =−0.45, respectively with p < 0.001 for both) according to Darahim et al. [37].

In the current study, RAVI and RVSP showed a significant positive correlation. Similar results were shown by Mantziari et al. [36] who uses inferior vena cava (IVC) diameter as a marker of increased central venous pressure and IVC dilatation. They noticed a strong correlation between RAVI and IVC diameter when volume overload of the right ventricle is present.

In the current study, RV diastolic function was evaluated using pulsed Doppler of the tricuspid inflow (tricuspid E/A) ratio and tricuspid annular TDI (tricuspid E/e') ratio, which revealed a significant correlation between RAVI and tricuspid E/A and E/e' ratios. However, Darahim [37], demonstrated that RAVI was not correlated with assessments of right ventricular diastolic dysfunction (tricuspid flow E/A ratio, or hepatic vein S/D ratio), which contradicts these results. These findings were also in contrast with those of Sallach et al. [34], who found a weak association between RAVI and the tricuspid flow E/A ratio and the hepatic vein S/D ratio.

The current study shows that inflammatory biomarkers might not only be useful to monitor treatment response but may also help to discriminate patients with a worsen prognosis. Hs-CRP, IL-1β and neopterin levels were higher in group I (CAT ≥ 10) in comparison to group II (CAT < 10), indicating different inflammatory response, while adiponectin concentrations tended to be lower in group I (CAT ≥ 10) in comparison to group II (CAT < 10). Lacoma et al. [39] showed higher CRP levels in patients with pneumonia compared to stable COPD and acute exacerbations of COPD, while significantly lower neopterin levels were only found in a small subgroup of acute exacerbations of COPD patients with bacterial isolates. Similarly, Chang et al. [40] described higher sensitive C-reactive protein (hs-CRP) levels at discharge as an independent predictor of readmission for acute exacerbations of COPD [40]. The relationships between CRP, IL6 and health related quality of life confirm the importance of systemic inflammation in patient perceptions of the impact and symptoms of COPD. In the same context, it was found that hs-CRP levels are higher in those patients with greater perception of breathlessness during mobilization [41]. Recent data show that CRP is an important predictor of future exacerbation and hospitalization [42].

Inconsistently, with our findings, a recent meta-analysis indicates that patients with COPD have higher serum adiponectin concentration than healthy controls [43]. Consistently with our results serum IL-1β may be an important biomarker for distinguishing patients with COPD from healthy subjects, which helps in evaluating the severity of COPD and predicting the clinical outcomes [44].

## Conclusion

The ability to visualize the right atrium allows a quantitative, highly reproducible assessment of the RA volume that can be indexed to body surface area (RAVI). In patients with chronic obstructive pulmonary disease, RAVI was significantly higher in patients with high CAT score and low functional capacity. During the usual evaluation of patients with COPD, an echocardiographic examination of the right side of the heart (RV systolic function and RAVI) may be helpful for identifying high risk patients. Additionally, our study provided evidence that biomarkers of inflammation hs-CRP, IL-1β, neopterin, and adiponectin may be helpful in the thorough evaluation and ongoing care of patients with COPD in primary care. As low adiponectin concentrations and high hs-CRP, IL-1β and neopterin levels, may help to discriminate patients with a worsen prognosis.

## Recommendations

Further studies are needed to confirm current data in larger population with different demographic and clinical characteristics and with an extended follow up duration.

## Limitations

Unfortunately, our study only covered one hospital (Tanta University hospitals), a relatively small number of patients, and mostly were male patients. Four chamber-views were used twice to compute the right atrium (RA) volumes using the biplane area length method (4C-4C). The right atrium lies in the distant field in this view, which reduces lateral resolution and makes it difficult to see the right atrial endocardium. Patients' functional ability was evaluated solely using the COPD Assessment Test (CAT) questionnaire, if we had included other functional tests, such as cardiopulmonary exercise, our findings might have been more reliable.

## Supplementary Information

Below is the link to the electronic supplementary material.

Supplementary file1 (PDF 68 KB)

Supplementary file2 (PDF 588 KB)

Supplementary file3 (PDF 235 KB)
