# Supplementary material for: Functional capacity and inflammatory biomarkers as predictors for right atrial volume index in COPD patients
Source: Int J Cardiovasc Imaging. 2023 May 22;39(8):1493–504. doi: 10.1007/s10554-023-02871-5 (PMC10427529; doi:10.1007/s10554-023-02871-5)
Supplement: Supplementary file 1 — Supplementary file1 (PDF 68 KB) [file 10554_2023_2871_MOESM1_ESM.pdf]

Highlights

- Chronic obstructive pulmonary disease (COPD) is a leading cause of mortality and right-heart complications.
- During the usual evaluation of patients with COPD, an echocardiographic examination of the right side of the heart (RV systolic function and RAVI) may be helpful for identifying high risk patients.
- Significantly higher RAVI was associated with high CAT score and low functional capacity in COPD Patients.
- Biomarkers of inflammation levels: Hs-CRP, IL-1β, neopterin, and adiponectin may be helpful in the thorough evaluation and ongoing care of patients with COPD in primary care.
