# Supplementary material for: Functional capacity and inflammatory biomarkers as predictors for right atrial volume index in COPD patients
Source: Int J Cardiovasc Imaging. 2023 May 22;39(8):1493–504. doi: 10.1007/s10554-023-02871-5 (PMC10427529; doi:10.1007/s10554-023-02871-5)
Supplement: Supplementary file 2 — Supplementary file2 (PDF 588 KB) [file 10554_2023_2871_MOESM2_ESM.pdf]

group	Sex	Hb	Weight	Hight	BMI	Age	QRS	MitralEA	MitralEe	TricuspidEA	TricuspidEe´
1	1	13.00	98.12	1.70	33.95	55	1	2.10	14.00	2.00	8.80
1	1	13.00	67.15	1.60	26.23	47	0	2.00	18.00	2.90	9.20
1	1	12.00	75.63	1.65	27.78	71	2	2.10	15.00	2.00	8.00
1	1	13.00	85.00	1.65	31.22	67	1	0.60	5.40	0.50	7.00
1	0	11.00	77.13	1.62	29.39	67	1	2.40	14.00	2.20	8.00
1	1	12.00	85.10	1.59	33.66	78	0	4.00	18.00	2.50	8.50
1	1	13.00	96.89	1.66	35.16	49	1	2.20	19.60	1.10	7.00
1	1	13.00	85.06	1.71	29.09	49	0	2.00	18.00	2.90	9.20
1	1	13.00	108.73	1.73	36.33	67	1	0.60	5.40	0.50	7.00
1	1	12.00	71.98	1.81	21.97	70	0	0.75	18.00	2.50	8.50
1	1	11.00	111.85	1.76	36.11	60	0	0.30	7.00	1.60	7.00
1	1	13.00	82.21	1.73	27.47	59	1	2.10	14.00	2.00	8.80
1	1	13.00	75.78	1.56	31.14	45	0	2.00	18.00	2.90	9.20
1	0	11.00	52.57	1.56	21.60	70	2	2.10	15.00	2.00	8.00
1	1	13.00	51.78	1.73	17.30	65	1	0.60	5.40	0.50	7.00
1	1	13.00	78.46	1.75	25.62	50	1	2.20	19.60	1.10	7.00
1	0	11.00	73.53	1.64	27.34	50	2	2.10	15.00	2.00	8.00
1	0	10.00	117.74	1.81	35.94	75	2	2.10	15.00	2.00	8.00
1	1	12.00	84.99	1.72	28.73	40	0	0.30	7.00	1.60	7.00
1	1	13.00	79.93	1.64	29.72	48	1	2.10	14.00	2.00	8.80
1	1	13.00	94.49	1.74	31.21	60	1	0.60	5.40	0.50	7.00
1	0	11.00	71.33	1.65	26.20	58	2	2.10	15.00	2.00	8.00
1	1	12.00	92.02	1.82	27.78	61	2	2.10	15.00	2.00	8.00
1	0	11.00	94.55	1.74	31.23	65	0	0.46	18.00	2.80	9.00
1	0	11.00	89.52	1.79	27.94	55	1	2.40	15.00	2.20	8.00
1	1	12.00	72.40	1.71	24.76	69	1	0.65	18.00	2.50	8.50
1	1	13.00	77.75	1.60	30.37	61	0	0.40	5.00	1.70	8.00
1	1	12.00	89.91	1.69	31.48	60	0	0.30	7.00	1.60	7.00
1	1	13.00	72.64	1.62	27.68	59	1	2.10	14.00	2.00	8.80
1	1	13.00	78.72	1.68	27.89	45	0	2.00	18.00	2.90	9.20
1	0	11.00	80.61	1.75	26.32	70	2	2.10	15.00	2.00	8.00
1	1	13.00	119.84	1.67	42.97	65	1	0.60	5.40	0.50	7.00
1	1	13.00	86.99	1.79	27.15	50	1	2.20	19.60	1.10	7.00
1	0	11.00	77.20	1.67	27.68	50	2	2.10	15.00	2.00	8.00
1	0	11.00	75.00	1.70	25.95	75	2	2.10	15.00	2.00	8.00
1	1	14.00	98.11	1.69	34.35	40	0	0.30	7.00	1.60	7.00
1	1	13.00	68.86	1.67	24.69	48	1	2.10	14.00	2.00	8.80
1	1	13.00	92.05	1.80	28.41	55	1	2.10	14.00	2.00	8.80
1	1	13.00	97.59	1.63	36.73	47	0	2.00	18.00	2.90	9.20
1	0	11.00	92.25	1.74	30.47	71	2	2.10	15.00	2.00	8.00
1	1	13.00	83.92	1.71	28.70	67	1	0.60	5.40	0.50	7.00
1	0	11.00	69.07	1.78	21.80	67	1	2.40	14.00	2.20	8.00
1	1	13.00	66.00	1.64	24.54	78	0	4.00	18.00	2.50	8.50
1	1	13.00	86.01	1.70	29.76	49	1	2.20	19.60	1.10	7.00
1	1	13.00	67.25	1.59	26.60	49	0	2.00	18.00	2.90	9.20
1	1	13.00	94.99	1.55	39.54	67	1	0.60	5.40	0.50	7.00
1	1	12.00	64.21	1.66	23.30	70	0	0.75	18.00	2.50	8.50
1	0	11.00	62.94	1.66	22.84	72	2	2.10	16.00	2.00	8.00
1	1	13.00	60.98	1.65	22.40	43	0	2.00	18.00	2.90	9.20

1	1	12.00	85.97	1.71	29.40	68	0	0.33	19.00	2.80	9.00
1	1	13.00	83.07	1.71	28.41	55	1	2.10	14.00	2.00	8.80
1	1	13.00	94.03	1.60	36.73	47	0	2.00	18.00	2.90	9.20
1	0	11.00	89.91	1.77	28.70	71	2	2.10	15.00	2.00	8.00
1	1	13.00	88.04	1.72	29.76	67	1	0.60	5.40	0.50	7.00
1	1	13.00	70.23	1.59	27.78	67	1	2.40	14.00	2.20	8.00
1	1	13.00	84.94	1.70	29.39	78	0	4.00	18.00	2.50	8.50
1	1	13.00	74.92	1.71	25.62	49	1	2.20	19.60	1.10	7.00
1	1	13.00	80.40	1.81	24.54	49	0	2.00	18.00	2.90	9.20
1	1	13.00	73.30	1.66	26.60	67	1	0.60	5.40	0.50	7.00
1	1	12.00	74.30	1.74	24.54	70	0	0.75	18.00	2.50	8.50
2	0	11.00	79.28	1.68	28.09	63	0	0.58	9.60	0.80	4.00
2	1	13.00	89.33	1.68	31.65	80	1	0.51	10.50	0.70	3.50
2	1	13.00	84.76	1.82	25.59	62	0	2.40	17.00	1.00	4.20
2	1	14.00	90.34	1.60	35.29	58	1	3.00	14.00	0.80	3.00
2	0	11.00	92.65	1.67	33.22	60	1	0.50	11.00	1.30	4.60
2	0	11.00	94.07	1.78	29.69	45	0	0.70	8.00	1.00	4.30
2	0	11.00	80.44	1.74	26.57	72	0	0.66	6.00	0.80	3.00
2	1	13.00	81.97	1.69	28.70	54	1	0.78	7.80	1.00	2.00
2	1	15.00	81.88	1.69	28.67	35	1	0.90	12.60	1.70	3.60
2	1	12.30	80.13	1.67	28.73	62	0	2.20	7.20	0.70	5.00
2	1	15.00	108.07	1.73	36.11	50	0	0.36	3.50	0.70	3.10
2	1	13.00	75.20	1.61	29.01	70	1	1.80	18.00	0.70	3.00
2	1	13.00	82.74	1.63	31.14	70	2	0.50	4.20	1.20	3.60
2	1	13.00	52.57	1.56	21.60	60	0	0.50	3.90	1.10	3.20
2	1	13.00	54.20	1.77	17.30	47	0	0.60	6.20	0.70	4.20
2	1	13.00	70.60	1.66	25.62	38	1	0.80	6.70	0.80	4.60
2	1	14.50	87.60	1.79	27.34	64	0	2.20	10.90	1.40	5.00
2	1	13.00	93.16	1.61	35.94	43	0	1.00	6.80	0.70	4.20
2	1	16.00	80.13	1.67	28.73	62	1	0.40	4.60	0.70	3.00
2	1	14.00	95.23	1.79	29.72	54	0	0.80	4.70	0.60	5.10
2	1	12.00	77.91	1.58	31.21	65	2	0.60	5.00	0.70	2.80
2	1	14.00	73.07	1.67	26.20	71	0	0.60	8.00	0.90	2.00
2	1	14.00	75.63	1.65	27.78	79	1	0.60	8.60	0.40	3.00
2	0	11.00	85.02	1.65	31.23	62	1	0.60	8.60	0.40	3.00
2	1	12.30	68.10	1.62	25.95	70	0	2.20	7.20	0.70	5.00
2	1	13.00	86.84	1.59	34.35	61	1	1.80	18.00	0.70	3.00
2	1	13.00	68.04	1.66	24.69	65	2	0.50	4.20	1.20	3.60
2	1	13.50	83.07	1.71	28.41	67	0	0.50	3.90	1.10	3.20
2	1	14.50	109.93	1.73	36.73	49	0	2.20	10.90	1.40	5.00
2	1	12.00	99.82	1.81	30.47	52	0	1.00	6.80	0.70	4.20
2	1	16.00	88.90	1.76	28.70	59	1	0.40	4.60	0.70	3.00
2	1	14.00	65.25	1.73	21.80	66	1	0.80	4.70	0.60	5.10
2	1	14.50	59.72	1.56	24.54	53	1	2.20	10.90	1.40	5.00
2	1	12.00	72.42	1.56	29.76	46	0	1.00	6.80	0.70	4.20
2	0	12.00	79.61	1.73	26.60	64	1	0.40	4.60	0.70	3.00
2	1	14.00	87.89	1.75	28.70	62	0	0.80	4.70	0.60	5.10
2	0	14.00	58.63	1.64	21.80	60	1	0.60	8.60	0.40	3.00
2	1	11.00	80.40	1.81	24.54	60	0	0.70	27.90	0.70	3.50
2	1	13.00	88.04	1.72	29.76	52	0	0.70	7.50	1.50	3.60

2	1	14.00	71.54	1.64	26.60	79	1	2.00	12.70	1.20	3.00
2	1	14.00	90.10	1.74	29.76	70	1	0.37	2.60	0.70	4.50
2	1	14.00	92.43	1.65	33.95	68	0	1.50	8.90	1.50	6.30
2	1	13.00	86.88	1.82	26.23	54	0	0.80	8.00	0.70	5.00
2	0	11.00	84.11	1.74	27.78	65	1	0.60	8.60	1.00	4.00
2	1	14.00	100.03	1.79	31.22	59	1	0.80	6.00	1.20	3.00
2	1	13.00	85.94	1.71	29.39	68	1	1.80	18.00	0.70	3.00
2	1	13.00	86.17	1.60	33.66	55	0	0.78	7.80	1.00	2.00
2	1	15.00	100.42	1.69	35.16	36	1	0.90	12.60	1.70	3.60
2	1	15.00	76.34	1.62	29.09	48	2	0.36	3.50	0.70	3.10
2	1	14.00	102.54	1.68	36.33	65	2	1.50	9.60	1.50	6.30
2	1	13.00	67.28	1.75	21.97	50	0	0.80	8.00	0.70	5.00
2	1	13.00	100.71	1.67	36.11	72	1	0.66	7.50	0.80	3.00
2	1	14.00	88.02	1.79	27.47	67	1	0.37	3.00	0.70	4.50
2	1	13.00	86.85	1.67	31.14	53	1	0.78	7.80	1.00	2.00
2	1	15.00	70.00	1.70	24.22	39	0	0.90	12.60	1.70	3.60
2	1	12.30	49.41	1.69	17.30	60	0	2.20	7.20	0.70	5.00
2	0	14.00	71.45	1.67	25.62	67	2	0.80	5.00	1.20	3.00
2	0	11.00	99.99	1.80	30.86	56	0	0.80	6.00	0.70	5.00
2	1	13.00	57.92	1.63	21.80	69	1	1.80	18.00	0.70	3.00
2	1	13.00	93.40	1.74	30.85	70	2	0.50	4.70	1.20	3.60
2	1	12.00	87.02	1.71	29.76	56	0	0.50	4.80	1.10	3.20
2	1	11.70	84.28	1.78	26.60	54	2	0.70	26.50	0.70	3.50
2	1	13.00	106.35	1.64	39.54	57	2	0.70	6.00	1.50	3.60
2	0	12.00	71.35	1.70	24.69	75	0	2.00	11.80	1.20	3.00
2	0	11.00	71.82	1.59	28.41	60	0	0.58	9.50	0.80	4.00
2	1	13.00	78.15	1.55	32.53	78	1	0.51	10.80	0.70	3.50
2	1	13.00	88.68	1.66	32.18	60	1	2.40	14.00	1.00	4.20
2	1	14.00	60.07	1.66	21.80	56	1	3.00	12.00	0.80	3.00
2	1	14.00	63.00	1.65	23.14	67	0	1.50	9.60	1.50	6.30
2	0	12.00	71.76	1.71	24.54	65	1	0.60	7.90	1.00	4.00
2	1	13.00	92.37	1.71	31.59	67	1	0.50	3.90	1.10	3.20
2	1	13.00	90.01	1.60	35.16	45	0	0.60	6.20	0.70	4.20
2	1	14.00	91.14	1.77	29.09	37	1	0.80	6.70	0.80	4.60
2	1	14.50	107.48	1.72	36.33	65	0	2.20	10.90	1.40	5.00
2	1	12.00	55.54	1.59	21.97	63	2	0.60	5.00	0.70	2.80
2	0	11.00	104.36	1.70	36.11	72	0	0.60	8.00	0.90	2.00
2	1	14.00	80.33	1.71	27.47	80	1	0.60	8.60	0.40	3.00
2	1	12.00	102.02	1.81	31.14	44	0	1.00	6.80	0.70	4.20
2	1	16.00	59.52	1.66	21.60	60	1	0.40	4.60	0.70	3.00
2	1	14.00	63.58	1.74	17.30	55	0	0.80	4.70	0.60	5.10
2	1	15.00	77.16	1.68	27.34	50	1	0.36	2.80	0.70	3.10
2	1	14.00	61.53	1.68	21.80	67	1	1.50	9.80	1.50	6.30
2	1	13.00	98.58	1.82	29.76	63	0	0.50	15.00	1.30	4.60
2	0	12.00	68.10	1.60	26.60	45	0	0.70	7.00	1.00	4.30
2	1	12.00	110.27	1.67	39.54	70	1	0.66	7.50	0.80	3.00
2	1	14.00	78.23	1.78	24.69	73	0	0.37	3.60	0.70	4.50
2	1	13.00	86.01	1.74	28.41	72	2	0.50	4.20	1.20	3.60
2	1	12.50	81.97	1.69	28.70	63	1	0.50	2.60	1.10	3.20
2	1	13.00	62.26	1.69	21.80	46	0	0.60	6.20	0.70	4.20
2	1	14.00	60.80	1.67	21.80	34	1	0.80	6.70	0.80	4.60
2	1	14.00	89.07	1.73	29.76	60	0	0.80	6.00	1.20	3.00

Smitms	Strims	LVEF	CAT	RVSP	RAVI	TAPSEcm	hsCRP	IL1b	Neoptrin	Adep	ACEI
0.08	0.05	63.00	20.70	55.00	44.00	1.60	6.10	50	69	4.70	1
0.05	0.04	58.00	39.70	60.00	121.00	1.00	2.00	46	68	1.50	0
0.05	0.05	56.90	28.20	55.00	60.00	1.30	1.90	31	82	4.00	1
0.06	0.05	67.00	36.70	49.00	75.00	1.10	1.15	30	78	3.20	0
0.07	0.06	59.00	36.90	64.00	45.00	1.20	1.30	30	93	5.00	1
0.05	0.06	68.00	38.20	56.00	62.00	1.10	1.12	31	104	4.00	0
0.05	0.05	58.90	38.00	67.00	66.00	1.10	1.12	33	86	5.40	0
0.05	0.08	58.00	39.70	60.00	99.00	1.00	6.00	51	75	1.60	0
0.06	0.05	56.00	36.70	48.00	75.00	1.10	3.98	36	88	2.60	0
0.05	0.05	58.00	38.90	50.00	44.00	1.20	1.15	30	96	4.30	0
0.06	0.05	67.00	28.70	38.00	65.00	1.30	3.50	35	108	2.00	0
0.08	0.05	63.00	20.70	60.00	66.00	1.60	2.00	33	83	2.00	1
0.05	0.05	58.00	36.70	45.00	75.00	1.00	3.90	70	110	2.50	1
0.05	0.05	57.50	28.70	60.00	60.00	1.20	1.77	31	97	3.50	0
0.06	0.05	68.00	36.70	45.00	75.00	1.10	3.90	68	85	2.00	0
0.05	0.05	58.90	38.00	57.00	90.00	1.10	4.10	70	105	1.61	1
0.05	0.05	57.50	28.70	60.00	60.00	1.20	3.80	38	116	3.88	1
0.05	0.05	57.50	28.70	60.00	60.00	1.20	3.90	41	98	3.90	0
0.06	0.05	67.00	28.70	38.00	65.00	1.30	3.70	35	65	3.50	0
0.08	0.05	63.00	20.70	60.00	66.00	1.60	3.90	65	98	3.50	1
0.06	0.05	68.00	36.70	45.00	75.00	1.10	4.00	61	77	2.10	1
0.05	0.05	57.50	28.70	60.00	60.00	1.20	2.60	40	105	3.93	1
0.05	0.05	57.50	28.70	60.00	60.00	1.20	3.30	39	73	3.95	0
0.05	0.05	68.00	28.80	50.00	44.00	1.20	1.10	28	122	4.60	1
0.07	0.05	59.00	36.70	60.00	75.00	1.20	4.00	55	81	2.10	0
0.05	0.07	68.00	38.20	55.00	87.00	1.30	1.30	25	104	2.72	0
0.06	0.08	67.00	38.70	40.00	89.00	1.20	1.30	26	87	2.80	0
0.06	0.05	67.00	28.70	38.00	65.00	1.30	3.50	50	78	3.95	0
0.08	0.05	63.00	20.70	60.00	66.00	1.60	2.00	30	90	3.90	1
0.05	0.05	58.00	36.70	45.00	75.00	1.00	3.90	55	66	3.50	1
0.05	0.05	57.50	28.70	60.00	60.00	1.20	3.90	55	69	4.15	0
0.06	0.05	68.00	36.70	45.00	75.00	1.10	4.10	60	117	0.80	0
0.05	0.05	58.90	38.00	57.00	121.00	1.10	6.10	70	69	1.10	1
0.05	0.05	57.50	28.70	60.00	60.00	1.20	2.10	38	82	4.00	1
0.05	0.05	57.50	28.70	60.00	60.00	1.20	3.90	41	99	4.00	0
0.06	0.05	67.00	28.70	38.00	65.00	1.30	3.70	35	128	4.40	0
0.08	0.05	63.00	20.70	60.00	66.00	1.60	3.90	35	132	4.40	1
0.08	0.05	63.00	20.70	55.00	44.00	1.60	1.10	31	74	4.70	1
0.05	0.04	58.00	39.70	60.00	121.00	1.00	6.10	70	87	1.50	0
0.05	0.05	56.90	28.20	55.00	60.00	1.30	1.15	24	96	4.90	1
0.06	0.05	67.00	36.70	49.00	75.00	1.10	2.30	30	104	3.90	0
0.07	0.06	59.00	36.90	64.00	45.00	1.20	3.10	30	85	4.30	1
0.05	0.06	68.00	38.20	56.00	85.00	1.10	5.60	60	77	2.50	0
0.05	0.05	58.90	38.00	67.00	83.00	1.10	5.00	55	68	2.40	0
0.05	0.08	58.00	39.70	60.00	120.00	1.00	6.10	70	105	1.60	0
0.06	0.05	68.00	36.70	48.00	75.00	1.10	1.98	26	103	2.60	0
0.05	0.05	68.00	38.90	50.00	85.00	1.20	5.00	55	65	3.30	0
0.05	0.05	58.00	36.70	65.00	75.00	1.30	1.10	24	87	3.80	1
0.05	0.06	58.00	39.20	70.00	120.00	0.90	2.18	31	93	1.60	0

0.05	0.05	67.50	29.80	50.00	55.00	1.20	1.00	23	96	3.50	1
0.08	0.05	63.00	20.70	55.00	44.00	1.60	2.00	31	78	4.70	1
0.05	0.04	58.00	39.70	60.00	120.00	1.00	2.40	35	115	1.50	0
0.05	0.05	56.90	28.20	55.00	60.00	1.30	1.10	23	108	3.90	1
0.06	0.05	67.00	36.70	49.00	75.00	1.10	1.00	34	132	3.90	0
0.07	0.06	59.00	36.90	64.00	73.00	1.20	2.50	30	88	3.30	1
0.05	0.06	68.00	38.20	56.00	82.00	1.10	3.40	37	72	2.30	0
0.05	0.05	58.90	38.00	67.00	80.00	1.10	3.00	31	94	2.40	0
0.05	0.08	58.00	39.70	60.00	121.00	1.00	6.00	71	98	1.60	0
0.06	0.05	68.00	36.70	48.00	75.00	1.10	2.20	28	72	2.10	0
0.05	0.05	68.00	38.90	50.00	86.00	1.20	3.15	33	102	3.60	0
0.08	0.16	60.00	4.60	17.00	18.00	1.80	1.00	20	76	6.30	0
0.06	0.13	59.00	8.50	20.00	21.00	2.60	1.20	19	56	7.10	0
0.07	0.14	68.00	3.60	55.00	22.00	1.90	1.22	22	76	6.80	0
0.05	0.09	63.70	9.95	69.00	32.00	1.50	2.58	21	62	4.82	1
0.05	0.16	67.20	5.80	32.00	17.00	1.90	2.16	26	84	7.40	0
0.07	0.13	67.80	6.20	31.00	17.00	1.80	1.60	18	86	7.50	0
0.09	0.13	64.00	7.95	40.00	30.00	2.50	2.00	21	74	4.50	0
0.05	0.15	71.50	2.80	25.00	17.00	1.70	2.00	21	86	7.63	0
0.10	0.10	69.00	2.60	28.00	18.00	2.20	1.20	26	75	7.40	0
0.08	0.05	56.20	9.95	46.00	27.00	2.80	3.55	70	68	4.90	1
0.07	0.07	68.00	3.40	15.00	16.00	1.80	1.00	24	77	8.40	0
0.08	0.17	61.00	9.95	30.00	29.00	1.50	2.90	25	68	4.60	1
0.08	0.11	67.00	2.70	35.00	16.00	1.90	3.90	27	92	8.40	0
0.08	0.11	63.00	4.50	35.00	18.00	1.70	2.00	20	84	7.20	1
0.06	0.13	56.00	5.60	22.00	22.00	1.60	3.70	28	68	6.80	0
0.06	0.14	72.00	2.40	10.00	16.00	2.70	3.90	28	89	8.50	0
0.08	0.13	70.00	2.40	35.00	16.00	2.00	3.90	28	102	8.80	0
0.08	0.12	67.00	4.50	15.00	19.00	2.20	3.80	26	73	6.95	0
0.05	0.13	64.60	4.70	15.00	20.00	2.00	2.50	22	58	5.30	0
0.08	0.11	63.90	2.90	22.00	17.00	2.00	3.50	27	64	7.77	1
0.08	0.13	60.00	9.50	36.00	32.00	2.20	3.90	28	62	4.10	0
0.08	0.19	62.00	8.70	25.00	29.00	3.00	3.80	39	89	4.30	0
0.05	0.11	65.00	7.40	27.00	21.00	2.00	3.90	30	66	6.90	0
0.05	0.11	65.00	7.40	27.00	21.00	2.00	2.10	33	105	6.90	0
0.08	0.15	56.20	9.95	46.00	27.00	3.00	1.70	23	69	5.33	0
0.08	0.17	61.00	9.95	30.00	27.00	2.50	3.80	39	83	5.50	1
0.08	0.11	67.00	2.70	35.00	16.00	1.90	3.40	30	71	7.80	0
0.08	0.11	63.00	4.50	35.00	23.00	2.10	1.60	20	58	5.30	0
0.08	0.13	70.00	2.40	35.00	15.00	2.00	3.80	35	68	7.99	1
0.08	0.12	67.00	4.50	15.00	23.00	2.20	3.90	27	55	4.90	0
0.05	0.13	74.60	4.70	15.00	25.00	2.00	2.40	21	52	4.60	1
0.08	0.11	73.90	2.90	22.00	18.00	1.60	1.10	16	102	7.00	0
0.08	0.13	70.00	2.40	35.00	19.00	2.00	2.60	23	85	6.20	1
0.08	0.12	67.00	4.50	15.00	24.00	2.20	3.30	25	88	5.20	0
0.05	0.13	64.60	4.70	15.00	25.00	2.00	3.00	21	78	4.50	0
0.08	0.11	63.90	2.90	22.00	16.00	1.60	3.10	28	130	7.90	0
0.05	0.11	65.00	7.40	27.00	21.00	2.00	2.50	21	125	6.30	0
0.06	0.11	75.00	5.40	15.00	19.00	1.90	2.78	30	86	7.50	0
0.08	0.15	73.00	6.50	25.00	20.00	1.90	2.24	24	92	7.10	0

0.08	0.13	67.80	8.60	15.00	25.00	2.10	2.49	30	78	4.60	0
0.06	0.11	62.00	6.65	35.00	20.00	1.80	1.00	20	96	5.52	0
0.07	0.17	73.00	9.95	11.00	31.00	3.00	1.00	18	88	3.20	0
0.06	0.15	61.00	7.25	14.00	26.50	2.70	3.80	31	67	4.50	0
0.05	0.11	65.00	6.90	27.00	19.00	2.20	2.30	25	65	7.00	1
0.08	0.12	67.00	9.20	24.00	30.00	2.90	2.20	26	110	3.60	0
0.08	0.16	62.00	9.95	35.00	31.00	3.00	1.78	19	96	3.60	0
0.05	0.20	71.50	2.60	32.00	16.00	1.50	2.24	21	69	7.80	1
0.10	0.11	69.00	2.60	20.00	16.00	2.30	1.49	18	82	7.80	1
0.07	0.18	65.00	3.40	18.00	18.00	2.00	2.00	25	102	7.40	1
0.07	0.09	73.00	9.85	30.00	35.00	3.10	2.10	20	89	3.50	0
0.06	0.14	61.00	7.40	35.00	25.80	2.80	2.20	20	65	5.18	0
0.09	0.14	64.00	7.85	22.00	24.00	2.50	1.90	18	98	5.70	1
0.06	0.10	65.00	6.65	22.00	20.00	1.80	2.60	19	110	6.00	1
0.05	0.17	71.50	2.80	28.00	16.00	1.80	1.60	19	128	7.45	1
0.10	0.11	69.00	2.70	35.00	16.00	2.40	1.50	28	89	7.50	1
0.08	0.15	76.20	9.95	37.00	31.00	3.00	2.00	25	83	3.80	0
0.08	0.12	66.00	9.75	25.00	30.00	1.80	2.39	27	88	4.00	1
0.06	0.06	61.00	7.25	30.00	26.50	3.00	1.80	19	102	5.05	0
0.08	0.08	66.00	9.95	30.00	30.00	1.60	0.97	19	79	4.30	1
0.08	0.12	64.00	2.70	36.00	16.00	2.10	0.85	19	98	7.50	1
0.08	0.11	62.00	4.56	25.00	24.00	1.70	1.11	22	68	5.90	1
0.06	0.12	77.00	5.60	24.00	26.00	1.80	0.97	19	56	3.80	1
0.08	0.15	73.00	6.50	30.00	27.00	1.90	0.65	17	66	3.90	0
0.08	0.14	67.80	8.60	25.00	29.00	2.20	0.81	18	67	4.00	0
0.08	0.16	60.00	4.60	23.00	24.00	2.30	0.89	18	58	5.10	0
0.06	0.14	59.00	8.50	20.00	28.00	2.00	0.74	18	60	4.10	1
0.07	0.13	68.00	3.60	36.00	17.00	2.60	0.66	16	63	7.00	0
0.05	0.09	63.70	9.95	33.00	34.00	1.50	1.12	26	70	3.80	0
0.07	0.07	73.00	9.95	10.00	48.00	3.40	1.15	27	69	2.50	0
0.05	0.12	65.00	6.90	36.00	25.00	2.00	0.85	17	71	4.20	0
0.08	0.12	63.00	4.50	38.00	18.00	1.90	0.64	16	66	6.10	0
0.06	0.14	76.00	5.75	26.00	20.00	1.60	1.31	15	67	4.30	0
0.06	0.15	72.00	2.40	22.00	18.00	2.70	1.13	30	68	6.40	0
0.08	0.13	70.00	2.60	28.00	18.00	2.10	1.42	26	66	6.40	0
0.08	0.14	80.00	9.50	30.00	32.00	2.50	0.95	18	59	4.10	0
0.08	0.20	62.00	8.70	25.00	31.00	3.00	0.99	18	60	4.60	0
0.05	0.11	65.00	7.40	27.00	27.00	2.90	1.11	20	63	5.80	0
0.08	0.13	67.00	4.50	30.00	18.00	2.20	0.71	17	67	6.70	1
0.05	0.12	64.60	4.70	22.00	19.00	2.00	1.13	34	68	6.20	0
0.08	0.11	63.90	2.85	15.00	16.00	1.50	1.10	33	67	7.10	1
0.07	0.17	65.00	3.40	15.00	17.00	1.80	0.99	17	77	6.60	0
0.07	0.15	73.00	9.95	10.00	33.00	2.90	0.76	17	65	4.10	0
0.05	0.18	67.20	5.80	24.00	17.00	2.30	0.46	16	66	6.80	0
0.07	0.14	67.80	6.20	32.00	17.00	1.70	1.01	19	72	6.50	0
0.09	0.13	64.00	7.95	40.00	28.00	2.60	0.91	17	65	4.10	0
0.06	0.11	65.00	6.65	23.00	22.00	1.80	0.84	20	57	5.90	0
0.08	0.12	67.00	2.70	25.00	15.00	1.90	0.74	18	67	7.40	0
0.08	0.11	63.00	4.50	22.00	18.00	1.70	0.88	18	66	6.90	1
0.06	0.13	65.00	5.60	18.00	20.00	1.60	0.90	19	68	5.10	0
0.06	0.16	72.00	2.40	22.00	18.00	2.70	0.70	18	69	6.70	0
0.08	0.11	67.00	9.20	22.00	33.00	3.20	1.40	23	63	6.80	1

ARB	BB	Statin	CCB	HTN	CAD	DM	Smoker	FH	SinusorAF	mMRC_class	Cr
0	0	0	0	1	0	0	1	0	1	3	1.00
1	0	0	1	0	0	1	1	1	1	2	1.20
0	0	1	0	1	1	1	0	0	0	3	0.90
0	0	1	0	0	1	0	0	0	0	2	0.80
0	0	0	0	1	0	1	0	0	0	2	0.90
1	0	0	0	0	0	0	0	0	1	2	0.90
1	0	1	0	1	1	0	0	1	0	3	1.00
0	0	0	0	0	0	0	0	0	0	1	1.20
0	0	0	0	0	0	0	0	1	0	1	0.80
0	0	0	0	0	0	0	1	0	1	2	1.10
1	1	1	0	1	1	1	1	0	0	2	1.20
0	1	0	0	0	0	1	1	0	0	3	1.00
0	1	1	0	1	1	1	1	1	1	1	1.20
1	0	0	1	0	0	1	0	1	1	1	0.90
1	0	0	0	1	0	1	0	0	0	3	0.80
0	0	0	0	0	0	1	1	0	0	2	1.00
0	0	0	0	0	0	1	0	0	0	1	0.90
1	0	1	0	1	1	1	1	1	0	1	0.90
1	0	0	0	1	0	1	1	0	0	1	1.20
0	0	0	0	0	0	1	0	0	0	3	1.00
0	0	0	0	1	0	1	1	1	0	2	0.80
0	0	1	0	0	1	1	0	1	0	1	0.90
1	0	0	1	0	0	1	1	1	0	1	0.90
0	0	0	0	1	0	1	1	0	1	2	0.90
1	0	0	0	1	0	1	1	1	0	2	0.90
0	0	0	0	0	0	0	0	0	0	3	0.90
0	0	0	1	0	0	0	0	0	0	3	1.20
1	1	1	0	1	1	1	1	0	0	1	1.20
0	1	0	0	0	0	1	1	0	0	1	1.00
0	1	1	0	1	1	1	1	1	0	1	1.20
1	0	0	1	0	0	1	0	1	0	3	0.90
1	0	0	0	1	0	1	0	0	0	2	0.80
0	0	0	0	0	0	1	1	0	0	3	1.00
0	0	0	0	0	0	1	0	1	0	1	0.90
1	0	1	0	1	1	1	1	1	0	1	0.90
1	0	0	0	1	0	1	1	0	0	3	1.20
0	0	0	0	0	0	1	0	0	0	3	1.00
0	0	0	0	1	0	0	1	0	0	3	1.00
1	0	0	1	0	0	1	1	1	1	2	1.20
0	0	1	0	1	1	1	0	1	0	1	0.90
0	0	1	0	0	1	0	1	0	1	1	0.80
0	0	0	0	1	0	1	1	0	0	2	0.90
1	0	0	0	0	0	0	0	0	1	3	0.90
1	0	1	0	1	1	0	0	1	0	2	1.00
0	0	0	0	0	0	0	1	1	0	2	1.20
0	0	0	0	0	0	0	1	0	0	1	0.80
0	0	0	0	0	0	0	1	0	1	1	1.10
0	0	1	0	1	1	0	0	1	0	3	0.90
1	0	0	1	1	0	0	1	1	0	3	1.20

0	1	1	0	1	1	1	0	0	0	1	0.90
0	0	0	0	1	0	0	1	0	0	3	1.00
1	0	0	1	0	0	1	1	1	0	3	1.20
0	0	1	0	1	1	1	0	1	0	1	0.90
0	0	1	0	0	1	0	1	1	0	1	0.80
0	0	0	0	1	0	1	1	1	0	3	0.90
1	0	0	0	0	0	0	0	0	1	3	0.90
1	0	1	0	1	1	0	1	1	0	3	1.00
0	0	0	0	0	0	0	1	1	0	2	1.20
0	0	0	0	0	0	0	1	1	0	2	0.80
0	0	0	0	0	0	0	1	0	1	3	1.10
1	0	0	0	1	0	1	0	0	0	1	1.00
1	0	0	0	1	0	1	0	0	0	2	1.70
0	0	1	0	1	1	0	0	1	0	3	0.90
0	1	1	0	1	1	1	1	0	0	3	1.00
1	0	0	0	0	0	1	1	1	0	1	1.00
0	0	1	0	0	1	0	1	1	0	1	1.20
0	0	0	0	0	0	0	1	0	0	2	1.20
0	0	0	0	0	0	0	0	0	0	1	1.00
0	0	0	0	0	0	0	0	0	0	1	0.90
0	0	1	0	1	1	1	1	0	0	2	2.00
0	0	0	0	1	0	0	0	1	0	1	1.00
0	0	0	0	1	0	1	1	0	0	2	1.20
1	0	0	0	0	0	0	1	0	0	3	0.80
0	1	1	1	0	1	0	0	0	1	3	1.00
1	0	0	0	1	0	0	0	1	1	1	1.40
1	0	0	0	0	0	0	1	1	0	1	1.10
1	0	0	0	1	0	0	1	0	0	3	1.10
1	0	0	0	0	0	0	1	1	0	3	0.90
1	1	1	0	0	1	1	1	0	1	1	1.20
0	0	0	0	0	0	0	1	0	0	1	0.90
0	0	1	0	0	1	0	1	0	0	2	0.80
1	0	0	1	0	0	0	0	1	0	2	1.40
0	0	0	0	0	0	0	0	0	0	1	1.00
0	0	0	0	0	0	0	0	0	0	1	1.00
1	0	0	1	1	0	1	0	0	0	2	2.00
0	1	1	1	1	1	1	1	0	0	2	1.20
0	0	0	0	0	0	0	1	0	0	3	0.80
1	0	0	0	0	0	0	1	0	0	3	1.00
0	0	0	0	1	0	0	1	0	0	1	1.10
1	0	1	0	0	1	0	1	1	0	1	0.90
0	0	0	1	0	0	1	1	0	0	3	1.20
0	0	1	0	0	1	0	1	0	0	3	0.90
0	0	0	0	1	0	0	1	0	0	3	1.10
0	0	0	0	0	0	0	1	1	0	1	0.90
1	0	0	0	0	0	1	1	0	0	1	1.20
0	0	0	0	0	0	0	1	1	0	3	0.90
0	0	0	0	0	0	0	0	0	0	3	1.00
1	0	0	0	1	0	0	0	0	0	1	1.00
0	0	1	0	0	1	0	0	0	0	1	1.00

1	0	0	0	1	0	0	0	1	0	2	1.00
1	0	0	0	1	0	1	1	0	0	2	1.00
0	0	1	0	1	1	0	0	0	0	2	1.00
1	0	0	0	1	0	1	1	1	0	2	0.90
0	0	0	0	0	0	1	1	0	0	1	1.00
1	0	0	0	1	0	1	0	1	1	1	0.90
1	0	0	0	0	0	1	0	0	0	2	1.20
0	0	1	0	1	1	1	1	1	0	3	1.00
0	1	0	0	1	0	1	1	0	0	3	0.90
0	0	0	0	0	0	0	1	1	0	1	1.00
1	0	0	0	0	0	1	1	0	0	2	1.30
1	0	0	0	0	0	0	1	1	0	2	0.90
0	0	1	1	1	1	1	0	1	0	2	1.20
0	1	1	0	0	1	0	1	1	1	2	1.00
0	1	0	0	1	0	1	0	0	0	1	1.00
0	0	0	0	1	0	1	0	0	0	1	0.90
0	0	0	0	0	0	0	1	0	0	2	2.00
0	0	0	0	1	0	1	1	1	0	1	0.90
0	0	0	0	0	0	0	1	1	0	2	0.90
0	0	1	0	0	1	1	1	1	0	2	1.20
0	0	0	0	1	0	1	1	1	0	3	0.80
0	0	0	0	1	0	1	0	1	0	3	1.00
0	0	0	0	1	0	0	0	1	0	1	1.00
1	0	0	0	1	0	0	1	1	0	1	0.90
1	0	0	0	0	0	1	1	1	0	2	1.00
1	0	0	0	1	0	0	1	0	0	1	1.00
0	0	1	0	0	1	1	1	0	0	2	1.70
1	0	0	0	1	0	1	0	1	0	1	0.90
1	0	0	0	1	0	0	1	1	0	3	1.00
1	1	0	0	1	0	1	0	1	0	2	1.30
0	1	1	0	0	1	1	1	0	0	1	1.00
1	0	0	0	1	0	1	1	0	0	3	1.00
0	0	1	0	0	1	0	0	1	0	3	1.40
1	0	0	0	1	0	1	0	1	0	3	1.10
1	0	0	0	1	0	0	1	0	0	1	1.10
1	0	0	0	0	0	1	1	0	1	2	0.80
0	0	1	0	0	1	0	1	1	1	2	1.40
1	0	0	0	1	0	0	1	0	0	1	1.00
0	0	0	0	1	0	0	1	1	0	1	0.90
0	0	0	0	0	0	0	1	0	0	3	1.20
0	0	0	0	1	0	0	0	0	1	3	0.90
1	0	0	0	0	0	1	0	1	1	1	1.00
0	0	0	0	0	0	0	1	0	0	2	1.30
1	0	0	0	1	0	0	1	1	0	1	1.00
1	0	1	0	1	1	1	1	1	0	1	1.20
1	0	0	0	0	0	1	0	1	0	2	1.20
1	0	1	0	0	1	1	1	1	1	2	1.00
1	0	0	0	1	0	0	0	1	1	3	0.80
0	0	0	0	1	0	0	1	1	1	3	1.00
1	0	0	0	1	0	0	1	1	1	3	1.40
0	0	1	0	0	1	0	1	1	0	1	1.10
0	1	0	0	1	0	1	1	1	0	1	0.90

Cholesterol	HbA1C	SBP	DBP	FVC	FEV1s	PaO2	FEV1/FVC
168	6.0	130.00	80.00	2.60	1.64	78.00	0.63
170	6.0	131.00	90.00	2.90	1.56	67.00	0.54
230	6.0	140.00	85.00	3.30	1.40	73.00	0.42
205	7.9	145.00	90.00	3.10	1.50	69.00	0.48
184	6.4	133.00	85.00	3.16	1.50	69.00	0.47
186	5.8	138.00	80.00	3.00	1.40	68.00	0.47
195	6.7	140.00	90.00	3.00	1.30	68.00	0.43
195	6.4	125.00	75.00	2.80	1.20	67.00	0.43
178	6.7	130.00	80.00	2.10	1.20	70.00	0.57
175	7.1	140.00	85.00	2.30	1.40	68.00	0.61
195	6.7	133.00	80.00	2.50	1.30	73.00	0.52
235	6.5	135.00	80.00	2.70	1.60	78.00	0.59
188	5.7	138.00	80.00	2.39	1.50	69.00	0.63
194	5.6	140.00	90.00	2.50	1.50	74.00	0.60
183	5.9	145.00	80.00	2.41	1.40	70.00	0.58
177	5.5	150.00	90.00	2.39	1.50	68.00	0.63
240	5.0	130.00	80.00	2.35	1.45	75.00	0.62
190	6.1	132.00	80.00	2.34	1.50	75.00	0.64
177	5.9	120.00	85.00	2.70	1.50	75.00	0.56
188	5.7	125.00	80.00	3.02	1.70	78.00	0.56
225	5.5	130.00	80.00	2.10	1.41	68.00	0.67
178	6.5	131.00	79.00	2.60	1.50	73.00	0.58
189	5.8	135.00	80.00	2.60	1.30	73.00	0.50
183	5.7	130.00	80.00	3.19	1.40	73.00	0.44
182	5.5	130.00	80.00	3.06	1.50	69.00	0.49
185	7.4	140.00	80.00	3.00	1.50	68.00	0.50
190	7.4	145.00	80.00	3.00	1.50	68.00	0.50
195	6.7	130.00	80.00	2.95	1.40	73.00	0.47
235	6.5	135.00	80.00	3.41	1.50	78.00	0.44
188	5.7	136.00	80.00	3.10	1.50	69.00	0.48
194	5.6	139.00	81.00	3.00	1.50	74.00	0.50
183	5.9	140.00	85.00	2.86	1.10	69.00	0.38
177	5.5	133.00	80.00	2.94	1.20	68.00	0.41
240	5.0	135.00	80.00	2.99	1.50	73.00	0.50
190	6.1	140.00	85.00	3.42	1.60	73.00	0.47
177	5.9	133.00	80.00	3.09	1.70	73.00	0.55
188	5.7	135.00	80.00	3.60	1.50	78.00	0.42
168	7.0	130.00	80.00	3.60	1.60	77.00	0.44
170	6.0	135.00	80.00	2.90	1.60	65.00	0.55
230	6.0	136.00	80.00	3.30	1.40	72.00	0.42
205	7.9	139.00	81.00	3.01	1.30	69.00	0.43
184	6.4	130.00	80.00	2.95	1.50	68.00	0.51
186	5.8	130.00	80.00	3.00	1.60	67.00	0.53
195	6.7	140.00	80.00	3.00	1.50	67.50	0.50
195	6.4	145.00	80.00	2.90	1.40	65.00	0.48
178	6.7	130.00	80.00	3.10	1.50	65.10	0.48
175	7.1	130.00	80.00	3.00	1.50	68.00	0.50
240	8.1	140.00	80.00	3.10	1.50	69.00	0.48
180	7.0	145.00	80.00	2.90	1.60	65.00	0.55

180	7.0	132.00	80.00	3.30	1.60	72.00	0.48
168	8.0	120.00	85.00	2.60	1.50	78.10	0.58
170	6.0	125.00	80.00	2.90	1.40	65.00	0.48
230	6.0	130.00	80.00	3.30	1.45	73.00	0.44
205	7.9	132.00	80.00	3.10	1.50	69.00	0.48
184	6.4	120.00	85.00	3.10	1.60	68.00	0.52
186	5.8	125.00	80.00	2.90	1.50	67.00	0.52
195	6.7	130.00	80.00	2.95	1.50	67.10	0.51
195	6.4	132.00	80.00	2.90	1.40	65.00	0.48
178	6.7	120.00	85.00	3.10	1.70	68.10	0.55
175	7.1	155.00	95.00	2.90	1.50	67.00	0.52
214	5.7	130.00	80.00	3.32	1.99	80.00	0.60
177	5.6	135.00	80.00	3.10	1.68	79.00	0.54
193	8.2	138.00	80.00	3.40	1.77	85.00	0.52
196	7.7	140.00	90.00	3.08	1.40	79.00	0.45
178	7.3	145.00	80.00	3.30	2.00	82.00	0.61
245	8.3	150.00	90.00	3.06	1.80	81.00	0.59
216	5.6	130.00	80.00	3.00	1.90	79.50	0.63
188	5.8	132.00	80.00	3.90	1.70	85.00	0.44
175	6.8	120.00	85.00	4.00	1.88	86.00	0.47
180	6.5	125.00	80.00	3.05	1.40	79.00	0.46
283	7.2	130.00	80.00	3.67	2.00	85.00	0.54
186	6.5	131.00	79.00	3.00	1.40	78.50	0.47
179	5.4	135.00	80.00	3.90	2.10	87.00	0.54
192	5.7	130.00	80.00	3.30	2.20	85.00	0.67
186	8.8	130.00	80.00	3.26	1.90	83.00	0.58
215	5.7	140.00	80.00	4.00	1.80	87.00	0.45
175	5.6	145.00	80.00	4.01	1.66	87.00	0.41
218	5.6	130.00	80.00	3.30	1.70	85.00	0.52
196	5.6	135.00	80.00	3.28	1.80	85.00	0.55
192	6.6	138.00	80.00	3.83	1.70	90.00	0.44
172	5.9	140.00	90.00	2.63	1.43	77.00	0.54
169	6.1	145.00	80.00	2.94	1.76	77.00	0.60
220	6.2	150.00	90.00	3.05	1.88	78.00	0.62
230	6.0	130.00	80.00	3.06	1.78	78.00	0.58
210	5.6	132.00	80.00	2.63	1.37	75.00	0.52
185	5.5	120.00	85.00	2.63	1.40	75.00	0.53
215	6.0	125.00	80.00	3.82	1.89	85.00	0.49
190	5.9	130.00	80.00	3.30	1.98	86.00	0.60
190	6.4	131.00	79.00	3.89	1.96	88.00	0.50
200	5.4	135.00	80.00	3.30	1.68	84.00	0.51
170	6.0	130.00	80.00	3.25	1.88	84.00	0.58
170	5.6	130.00	80.00	3.69	1.99	86.00	0.54
186	7.1	140.00	80.00	3.82	2.00	87.00	0.52
228	6.4	145.00	80.00	3.26	1.80	80.00	0.55
196	8.0	130.00	80.00	3.20	1.77	80.00	0.55
234	6.4	135.00	80.00	3.89	1.80	85.00	0.46
180	7.7	138.00	80.00	3.05	1.81	75.00	0.59
196	6.7	140.00	90.00	3.24	1.86	79.00	0.57
192	5.5	145.00	80.00	3.10	1.90	77.00	0.61

225	8.2	150.00	90.00	2.93	1.90	73.00	0.65
252	6.8	130.00	80.00	3.20	1.80	77.00	0.56
194	6.4	132.00	80.00	2.52	1.78	75.00	0.71
280	5.1	120.00	85.00	3.10	1.70	75.00	0.55
242	7.9	125.00	80.00	3.15	1.70	77.00	0.54
178	6.8	130.00	80.00	2.52	1.90	70.00	0.75
185	7.4	131.00	79.00	2.51	1.99	70.00	0.79
265	8.8	135.00	80.00	3.90	1.80	82.00	0.46
190	7.0	130.00	80.00	3.88	2.00	82.00	0.52
188	7.2	130.00	80.00	3.57	2.10	80.00	0.59
175	8.1	140.00	80.00	2.41	2.00	70.00	0.83
188	6.9	145.00	80.00	3.04	1.90	74.00	0.63
287	6.0	130.00	80.00	3.00	1.99	73.00	0.66
210	7.0	135.00	80.00	3.15	1.96	78.00	0.62
220	7.9	138.00	80.00	3.89	1.95	85.00	0.50
194	6.0	140.00	90.00	4.69	1.90	86.00	0.41
195	6.5	145.00	80.00	2.41	1.36	70.00	0.56
180	7.3	150.00	90.00	2.51	1.40	71.00	0.56
215	7.9	130.00	80.00	3.13	1.67	78.00	0.53
193	7.0	132.00	80.00	2.80	1.40	70.00	0.50
220	7.0	120.00	85.00	3.62	1.80	85.00	0.50
172	6.0	125.00	80.00	3.16	1.80	84.00	0.57
190	6.5	130.00	80.00	3.05	1.70	83.00	0.56
180	7.3	131.00	79.00	3.05	1.70	82.00	0.56
220	7.0	135.00	80.00	2.62	1.80	76.00	0.69
210	6.0	130.00	80.00	3.30	1.70	83.00	0.52
194	7.9	130.00	80.00	2.52	1.70	76.00	0.67
192	7.1	140.00	80.00	3.40	1.60	84.00	0.47
228	6.4	145.00	80.00	2.51	1.70	71.00	0.68
205	8.0	130.00	80.00	2.51	1.70	71.00	0.68
182	6.5	135.00	80.00	3.15	1.80	76.00	0.57
197	7.7	138.00	80.00	3.46	1.80	85.00	0.52
200	6.7	140.00	90.00	3.20	1.70	83.00	0.53
180	5.5	145.00	80.00	3.88	2.00	87.00	0.52
178	7.1	150.00	90.00	3.86	2.00	86.00	0.52
176	6.4	130.00	80.00	2.51	1.40	71.00	0.56
192	8.0	132.00	80.00	2.65	1.80	72.00	0.68
180	6.4	120.00	85.00	3.00	1.70	73.00	0.57
200	6.5	125.00	80.00	3.36	1.80	82.00	0.54
210	5.8	130.00	80.00	3.46	1.80	81.00	0.52
183	7.7	131.00	79.00	4.09	2.20	86.00	0.54
198	5.5	135.00	80.00	4.02	2.00	83.00	0.50
180	7.1	130.00	80.00	2.81	1.40	70.00	0.50
160	8.0	130.00	80.00	3.65	1.80	85.00	0.49
148	6.4	140.00	80.00	3.24	1.80	83.00	0.56
185	6.5	145.00	80.00	3.13	1.50	68.00	0.48
192	5.8	130.00	80.00	3.24	1.57	80.00	0.48
172	7.7	135.00	80.00	4.09	2.12	87.00	0.52
198	5.5	130.00	80.00	3.65	1.79	81.00	0.49
186	7.0	130.00	80.00	3.35	1.69	80.00	0.50
200	6.7	140.00	80.00	3.99	2.12	87.00	0.53
170	7.2	145.00	80.00	2.90	1.40	70.00	0.48
