# Supplementary material for: Functional capacity and inflammatory biomarkers as predictors for right atrial volume index in COPD patients
Source: Int J Cardiovasc Imaging. 2023 May 22;39(8):1493–504. doi: 10.1007/s10554-023-02871-5 (PMC10427529; doi:10.1007/s10554-023-02871-5)
Supplement: Supplementary file 3 — Supplementary file3 (PDF 235 KB) [file 10554_2023_2871_MOESM3_ESM.pdf]

Supplementary Figure: Chronic Obstructive Pulmonary Disease (COPD) Assessment Test (CAT).[1]

Your name:

Today's date:

CAT

How is your COPD? Take the COPD Assessment Test™ (CAT)

This questionnaire will help you and your healthcare professional measure the impact COPD (Chronic Obstructive Pulmonary Disease) is having on your wellbeing and daily life. Your answers, and test score, can be used by you and your healthcare professional to help improve the management of your COPD and get the greatest benefit from treatment.

For each item below, place a mark (X) in the box that best describes you currently. Be sure to only select one response for each question.

Example: I am very happy 0 X 2 3 4 5 I am very sad

			SCORE
I never cough	0 1 2 3 4 5	I cough all the time	
I have no phlegm (mucus) in my chest at all	0 1 2 3 4 5	My chest is completely full of phlegm (mucus)	
My chest does not feel tight at all	0 1 2 3 4 5	My chest feels very tight	
When I walk up a hill or one flight of stairs I am not breathless	0 1 2 3 4 5	When I walk up a hill or one flight of stairs I am very breathless	
I am not limited doing any activities at home	0 1 2 3 4 5	I am very limited doing activities at home	
I am confident leaving my home despite my lung condition	0 1 2 3 4 5	I am not at all confident leaving my home because of my lung condition	
I sleep soundly	0 1 2 3 4 5	I don't sleep soundly because of my lung condition	
I have lots of energy	0 1 2 3 4 5	I have no energy at all	
		TOTAL SCORE	

[1]Mackay AJ, Donaldson GC, Patel AR, Jones PW, Hurst JR, Wedzicha JA. Usefulness of the Chronic Obstructive Pulmonary Disease Assessment Test to evaluate severity of COPD exacerbations. Am J Respir Crit Care Med. 2012;185(11):1218-24. doi: 10.1164/rccm.201110-1843OC. PMID: 22281834.
